# Immobilization of β-Glucosidase from *Thermatoga maritima* on Chitin-functionalized Magnetic Nanoparticle via a Novel Thermostable Chitin-binding Domain

**DOI:** 10.1038/s41598-019-57165-5

**Published:** 2020-02-03

**Authors:** Fawze Alnadari, Yemin Xue, Liang Zhou, Yahya S. Hamed, Mohamed Taha, Mohamed F. Foda

**Affiliations:** 1 Department of Food Science and Engineering, School of Food Science and Pharmaceutical Engineering, Nanjing Normal University, Nanjing, 210023 Jiangsu, P.R. China; 2 State Key Laboratory of Agricultural Microbiology, College of Science, Huazhong Agricultural University, Wuhan, 430070 P. R. China; 3 State Key Laboratory of Agricultural Microbiology, College of Veterinary Medicine, Huazhong Agricultural University, Wuhan, 430070 P. R. China; 4 Department of Biochemistry, Faculty of Agriculture, Benha University, Moshtohor, Toukh, 13736 Egypt; 5 Centre for Environmental Sustainability and Remediation, RMIT University, Bundoora, Melbourne, VIC 3083 Australia; 6 Department of Food Science and Technology, Faculty of Agriculture, Sana’a University, Sana’a, Yemen; 7 Food Technology Department, Faculty of Agriculture, Suez Canal University, Ismailia, 41522 Egypt

**Keywords:** Magnetic properties and materials, Carbohydrates, Enzymes

## Abstract

Enzyme immobilization is a powerful tool not only as a protective agent against harsh reaction conditions but also for the enhancement of enzyme activity, stability, reusability, and for the improvement of enzyme properties as well. Herein, immobilization of β-glucosidase from *Thermotoga maritima* (Tm-β-Glu) on magnetic nanoparticles (MNPs) functionalized with chitin (Ch) was investigated. This technology showed a novel thermostable chitin-binding domain (Tt-ChBD), which is more desirable in a wide range of large-scale applications. This exclusive approach was fabricated to improve the Galacto-oligosaccharide (GOS) production from a cheap and abundant by-product such as lactose through a novel green synthesis route. Additionally, SDS-PAGE, enzyme activity kinetics, transmission electron microscopy (TEM) and Fourier transform infrared spectroscopy (FT-IR) revealed that among the immobilization strategies for *Thermotoga maritime*-β-Glucosidase thermostable chitin-binding domain (Tm-β-Glu-Tt-ChBD) on the attractive substrate; Ch-MNPs had the highest enzyme binding capacity and GOS production ratio when compared to the native enzyme. More interestingly, a magnetic separation technique was successfully employed in recycling the immobilized Tm-β-Glu for repetitive batch-wise GOS without significant loss or reduction of enzyme activity. This immobilization system displayed an operative stability status under various parameters, for instance, temperature, pH, thermal conditions, storage stabilities, and enzyme kinetics when compared with the native enzyme. Conclusively, the GOS yield and residual activity of the immobilized enzyme after the 10^th^ cycles were 31.23% and 66%, respectively. Whereas the GOS yield from native enzyme synthesis was just 25% after 12 h in the first batch. This study recommends applying Tt-ChBD in the immobilization process of Tm-β-Glu on Ch-MNPs to produce a low-cost GOS as a new eco-friendly process besides increasing the biostability and efficiency of the immobilized enzyme.

## Introduction

Lactose is a low-priced disaccharide due to its low relatively sweetness (approximately 15% as sweet as sucrose) and commercially produced in large quantities as a by-product from dairy industry worldwide. Owing to the relatively low solubility of lactose in most solvents, which makes this compound undesirable in various industrial applications^1^. Additionally, lactose intolerance is a gastric disorder caused by the incapability of human guts to digest milk sugar that widespread in the world population. Subsequently, lactose exemplifies a significant carbohydrate supply for industrialized procedures on value-added products to endorse substaional enzymatic development. One imperative submission of lactose conversion is the fabrication of compounds such as galacto-oligosaccharides (GOS). This profitable GOS products are then converted through a well-known transglycosylation reactions via retaining β-glucosidases (E.C. 3.2.1.21)^2^. GOS are also categorized as functional food ingredients and useful dietary fibers presenting a great interest in the field of enzyme reaction engineering and industrial interest in food related biotechnology^3^. Several commercial and non-commercial β-glucosidase (β-Glu) extracted from different sources (including microbial sources) have been evaluated under different temperature conditions for their ability and efficiency to produce GOS^4^.

*Thermotoga maritima* is a thermophilic, heterotrophic, halophilic, and obligate anaerobic bacterium that can produce β-Glu with a variety of different substrates, which can be applied in many industrial applications^5,6^. *T. maritima* is a thermophilic bacterium that grows typically in extreme conditions (between 60–90 °C) with an ideal growth temperature of 85 °C and is of considerable interest due to the hyper-thermostability of these enzymes (TmBglA & TmBglB). The TmBglA has a broad substrate specificity and belongs to glycoside hydrolase family 1 β-glucosidases (GH1), which make it ideal to be applied in numerous industrial tenders^7^. As reported, β-glucosidases from *T. maritima* (Tm-β-Glu) present an efficient catalyst to break the glycosidic bond with a high conversion percentage in thermal conditions^8^. Rational design and enzyme engineering improve the transglycosylation-to-hydrolysis ratio and produce more beneficial enzyme variants giving higher GOS production yield^2^. To assemble a chimeric protein which contain a functionalized catalytic domain via genetic engineering strategies, in conjunction with a binding-domain, has already proven to be beneficial, yet still encountering technical barriers. A remarkable enhancement of enzyme properties can be successfully achieved by using enzyme engineering technique in multiple catalytic methods. Furthermore, enzyme immobilization is one of the most widely used techniques, wherein catalysts are affixed to reliable support that is insoluble in the reaction mixture^9,10^.

Enzyme immobilization is a prominent tool that is cost-effective mainly when applied on a large scale due to enhancing enzyme stability, activity, and reusability. Additionally, immobilization process of enzymes is suitable for commercial applications aiming to suitability in handling. As well as privileged circumstances in separating enzymes from reaction mixtures, reuse, low production cost with the possibility of increasing thermal and pH stability^11,12^. Researchers are commissioning current technologies and developing modern applications that utilize immobilized enzymes using current technology of nanoparticles^13^. Presently, many enzymes used in biotechnology, including β-Glu, have been covalently immobilized to magnetic nanoparticles (MNPs) using numerous ligands^1,14,15^. After the enzyme molecules binding process, the catalysts transfer to a heterogeneous form simplifying the biocatalytic separation system from the reaction mixture and produce enzymnes with higher purity^16^. The nanoparticles physical characteristics, such as particle mobility and boost diffusion, can impact on the fundamental catalytic activity of attached enzymes^17^. On the other hand, the magnetic field capability reveals a mechanism for enzyme recovery efficiency, thereby preventing the enzyme contamination in the final products^1,18^.

Moreover, immobilization has related benefits for instance probability of constant usage, minimum reaction time, high stability, enhanced activity control, and incessant product division, as well as the enzyme stability and activity, must be satisfactory during the entire reaction mechanism^19^. Pimentel *et al*. (1991), reported that the immobilized starch-enzymes improved the starch hydrolysis when compared to a free enzyme under the same conditions (various temperature sets) and enhanced the thermal stability as well^20,21^.

**Figure 1:**
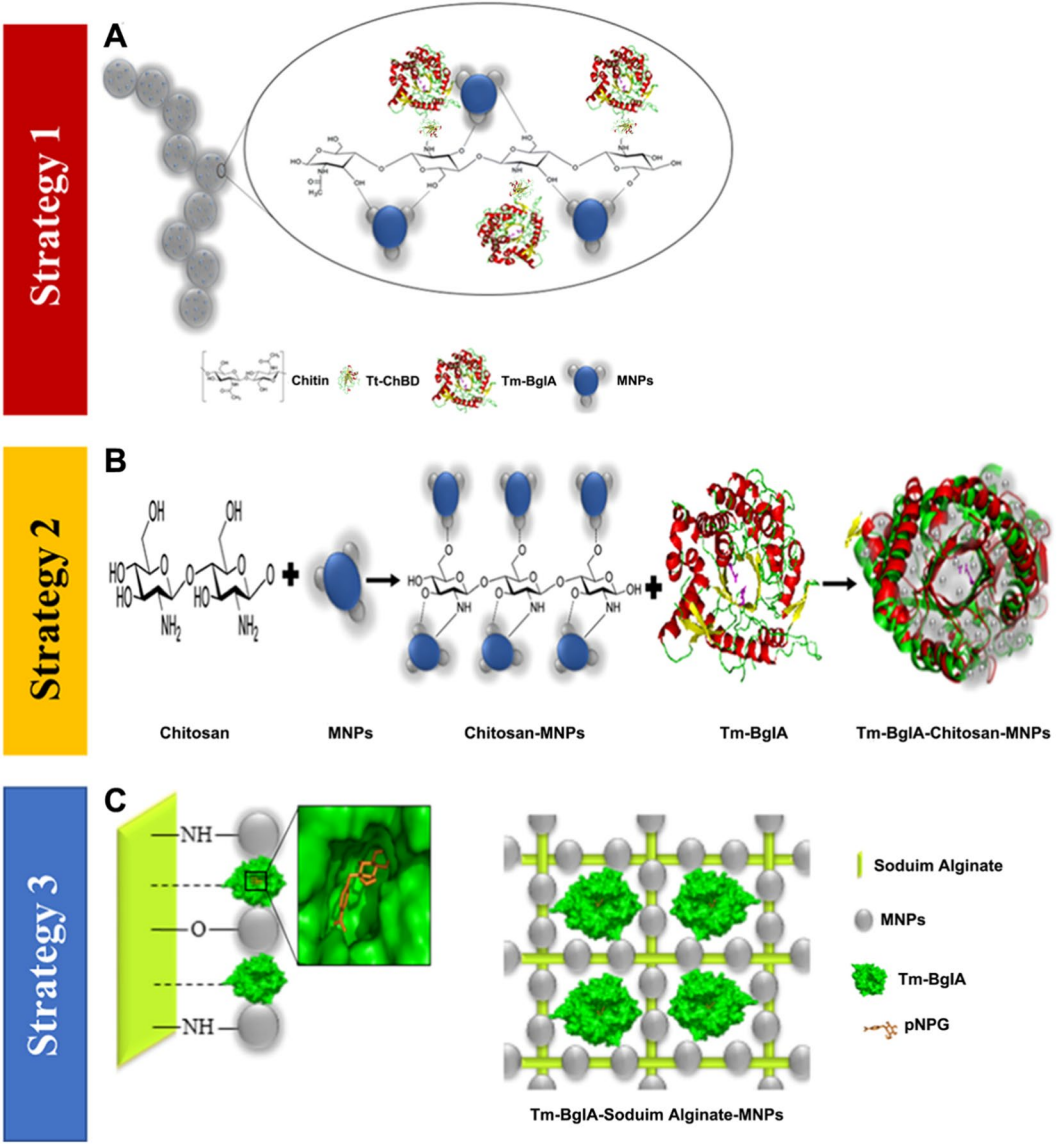
Schematic illustration of Tm-β-Glu-Tt-ChBD-Ch-MNPs, Tm-β-Glu-SA-MNPs and Tm-β-Glu-Cs-MNPs synthetic strategies of (**A**) chitin-iron oxide nanoparticles and Tm-β-Glu-Tt-ChBD immobilization route (**B**) chitosan-iron oxide nanoparticles and Tm-β-Glu immobilization route (**C**) sodium alginate-iron oxide nanoparticles and Tm-β-Glu immobilization route. The deduced amino acid sequences of the fusion proteins and chemical structure of three methods with MNPs were used to predict their 3D structure using the RaptorX tool (http://raptorx.uchicago.edu/).

Among the various available immobilization techniques, the approach of confining enzymes on chitin (Ch) by fusing target proteins with chitin-binding domain (ChBD) received considerable attention, mostly due to the desirable characteristics of the Ch supports for instance low-cost and accessibility, elevated stability to both temperature and pH, and microbial resistance^7,22,23^. Therefore, the application of applying Tm-β-Glu in the immobilized form with chitin-Fe_3_O_4_ magnetic nanoparticles (Ch-MNPs) by a novel thermostable chitin-binding domain (Tt-ChBD) will be more commercially advantageous. This study aimed to focus on the immobilization of Tm-β-Glu on MNPs functionalized with three biopolymers: chitin, chitosan, and sodium alginate with a novel Tt-ChBD as illustrated below in Fig. 1. Besides, this study investigated the GOS production from lactose through a green synthesis system. Moreover, the recombinant enzymes were fused to the Tt-ChBD via molecular tools. The iron-oxide magnetic nanoparticles were synthesized, and enzyme immobilization on the activated nanoparticle was carried out using a covalent binding method. Besides, reusability of the immobilized enzyme was similarly characterized concerning the number of batches of lactose hydrolysis, which increase the final yield of GOS production.

## Results and Discussion

### Production of recombinant Tm-β-Glu fused Tt-ChBD and Ch-MNPs binding

**Figure 2:**
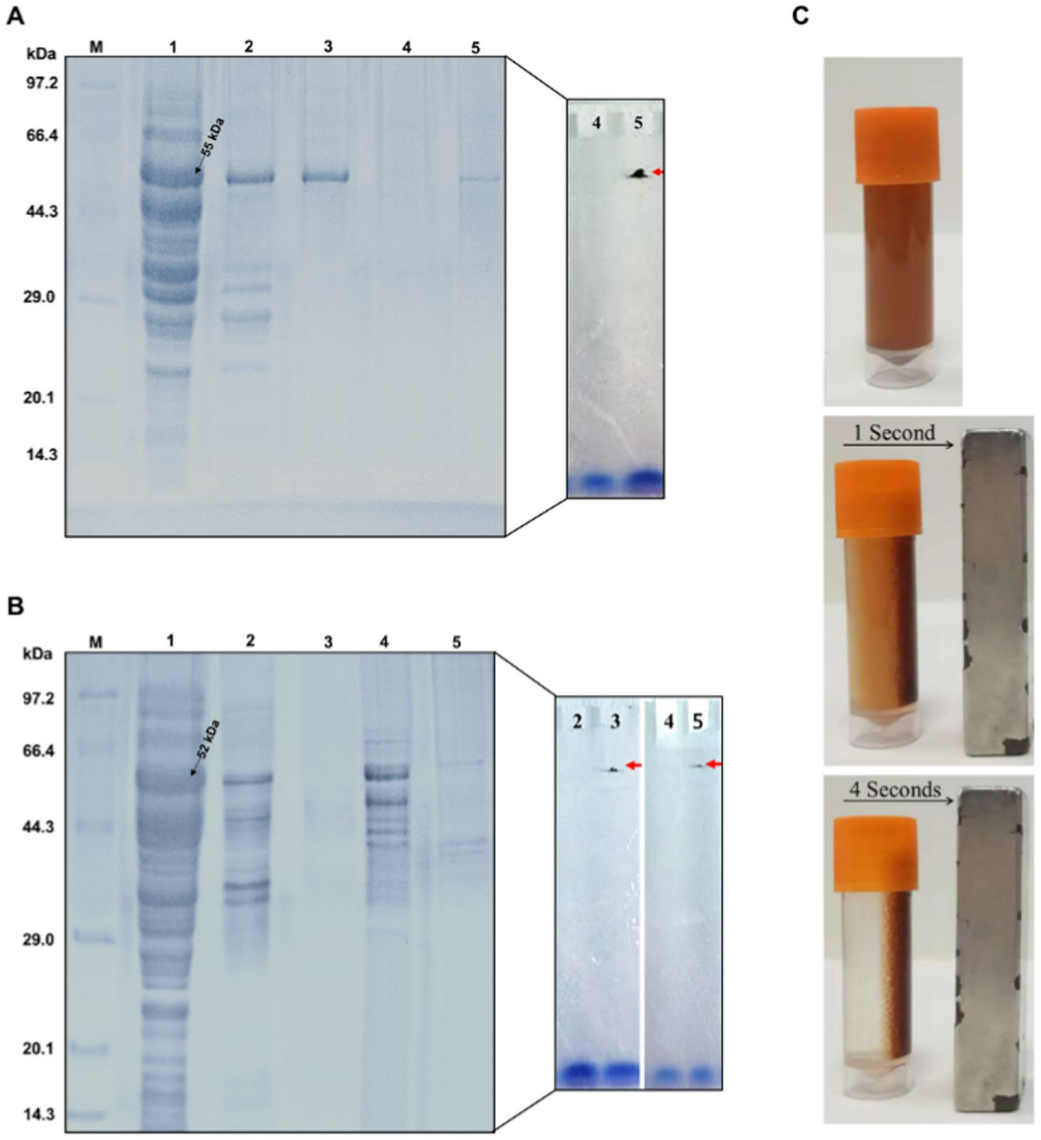
(**A**) SDS-PAGE analysis: total cell proteins of *E. coli* JM109 (DE3) harboring Tm-β-Glu-Tt-ChBD (lane 1A); After heat treatments (lane 2A); After purification (lane 3A); Non-immobilized supernatant β-Glu-Tt-ChBD on Ch-MNPs (lane 4A); Tm-β-Glu-Tt-ChBD-Ch-MNPs (lane 5A); (**B**) SDS-PAGE analysis: total cell proteins *E. coli* JM109 (DE3) harboring Tm-β-Glu (lane 1B); Non-immobilized supernatant β-Glu on sodium alginate-MNPs (lane 2B); Tm-β-Glu-Sodium alginate-MNPs (lane 3B); Non-immobilized supernatant Tm-β-Glu on chitosan-MNPs (lane 4B); Tm-β-Glu-Chitosan-MNPs (lane 5B); (**C**) Tm-β-Glu-Tt-ChBD on chitin-MNPs affinity was presented by a magnetic bar in four seconds. (Uncropped gel images in Fig. 2A,B are in the Supplementary Information Figs. S1 and S2).

Engineering studies on Tt-ChBD and the expression plasmid Tt-ChBD has explained the sequences of the recombinant fusion proteins^7^. Tm-β-Glu-Tt-ChBD was constructed and successfully expressed in *E. coli* JM109 (DE3). The β-Glu activity expressed was increased to 8.48 U mg^−1^ in the crude extracts from 4.99 U mg^−1^ in the pET-28a-BglA. The expression levels of the recombinant protein were remarkably different depending on the position of Tt-ChBD when fused to Tm-β-Glu. The SDS-PAGE analysis of cell-free extracts of *E. coli* JM109 (DE3) harboring pET-28a-23aa-BglA and pET-28a-β-Glu revealed two proteins were found at 55 kDa and 52 kDa, respectively. Figure 2A, (lanes1 Tm-β-Glu-Tt-ChBD), and Fig. 2B, (lanes1 Tm-β-Glu) shows the molecular weight and purity of the fusion proteins. In order to verify the binding abilities of Tt-ChBD fused Tm-β-Glu heparinases^7^, a study was started to compare between applying the free and immobilized Tm-β-Glu against chitin to improve the production yields. The results showed that the Tt-ChBD domain was well-separated from the main protein pET-28a-BglA (in case of Tm-β-Glu-Tt-ChBD), where Tm-β-Glu fused Tt-ChBD interacts more tightly with the chitin magnetic nanoparticles MNPs as verified in Fig. 2C and in the Supplementary Video.

### Magnetic Nanoparticles immobilization of Tm-β-Glu-Tt-ChBD on Ch, Cs, and SA

The immobilization yield is considered a vital parameter in the immobilization process, which can indicate how much of the applied protein is bounded to the carrier. In this work, the immobilization yield for Tm-β-Glu-Tt-ChBD on Ch-MNPs has the uppermost significant (P > 0.05) yield comparing with the other two immobilized samples (Tm-β-Glu-Cs-MNPs, and Tm-β-Glu-SA-MNPs). The yield of Tm-β-Glu-Tt-ChBD-Ch-MNPs was up to 100% (while enzyme activity of 0% was detected in the supernatant after magnetic nanoparticles immobilization). These results confirmed the synergetic attachment of Tm-β-Glu-Tt-ChBD-Ch enzyme when chitosan and sodium alginate were selected and bounded on MNPs. In contrast, the immobilization yield of Tm-β-Glu-SA-MNPs and Tm-β-Glu-Cs-MNPs were 26 and 21%, respectively, as presented in Table 1. The findings we report herein, suggested that the remarkable properties of Tt-ChBD can be applied in the immobilization of enzymes by genetic engineering techniques, especially at high reaction temperature. This corresponds to previously published results for *Thermatoga maritima* β-Glu immobilized onto a variety of supports chitin beads^13^. These values were further confirmed by SDS-PAGE analysis via samples of the culture supernatant and precipitate after immobilization of β-Glu on Ch-MNPs, Ch-MNPs, and SA-MNPs, as shown in Fig. 2A (lanes 4,5 for Ch-MNPs and Fig. 2B (lanes 2,4 for SA-MNPs and Cs-MNPs). After completing the adsorption procedure, chitin beads were recovered by magnetic, and the precipitate was analyzed by gel electrophoresis as shown in Fig. 2A (lane 5 for Tm-β-Glu-Tt-ChBD-Ch-MNPs), Fig. 2B (lanes 2,5 for Tm-β-SA-MNPs and Tm-β-Cs-MNPs). The above findings indicated that the magnetic nanoparticles were coated successfully on the chitin with high affinity bound against Tm-β-Glu-Tt-ChBD to chitin-magnetic nanoparticles. Therefore, all further analyses were performed on Tm-β-Glu-Tt-ChBD and the native enzyme as control.

**Table 1:** Expression, purification, and immobilizations of thermostable Tm-β-Glu from *E. coli* JM109 (DE3) harboring pET-28a-β-Glu.

Recombinant proteins	Expressed activity (U mg^−1^)	Residual activity of the supernatant (%)	Yield (%)	purification (fold)
Free β-Glu	4.99^a^	—	100^c^	1.0^a^
Tm-β-Glu-Tt-ChBD-Ch-MNPs	58.06^d^	0.01^a^	99.9^c^	11.6^d^
Tm-β-Glu-SA-MNPs	42.86^c^	26.69^c^	73.31^a^	8.9^c^
Tm-β-Glu-Cs-MNPs	38.13^b^	21.27^b^	78.73^b^	7.6^b^

^*^Activity was determined with 40 mM 4-Nitrophenyl-β-D-glucopyranoside (pNPG) as a substrate in 50 mM citrate buffer (pH 6.2) at 90 °C for 5 min.

### Characterization of free and immobilized Tm-β-Glu-Tt-ChBD enzymes on smart magnetic particles

**Figure 3:**
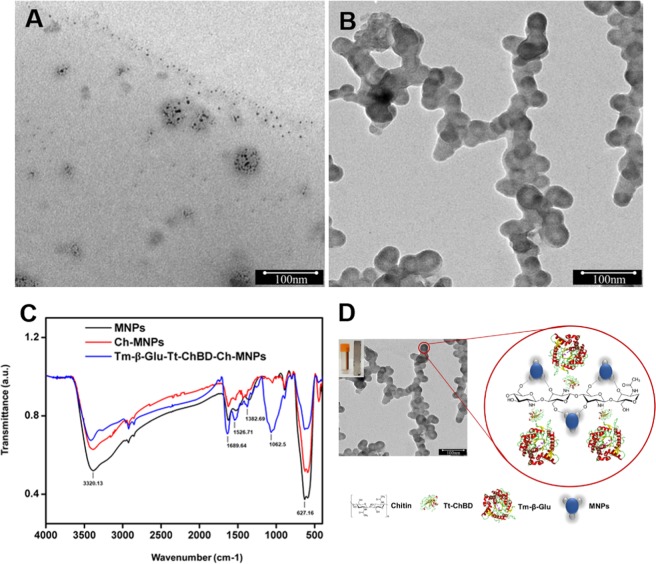
(**A**) TEM images of the pre-synthesized Fe_3_O_4_ MNPs, (**B**) Tm-β-Glu-Tt-ChBD-Ch-MNPs, (**C**) FTIR spectra, (**D**) Schematic structure of the functionalized Tm-β-Glu-Tt-ChBD-Ch-MNPs (inset the Tm-β-Glu-Tt-ChBD on chitin-MNPs affinity reclaimed by a magnetic bar).

Emerging a new catalytic system immobilized on magnetic nanocarriers received a significant deliberation nowadays due to the following key factors a) sizeable surface-to-volume ratio, b) high surface area, c) substantial superparamagnetism and d) no external diffusion problems, enabling multiple enzyme molecules to be immobilized on a single nanoparticle besides making them ideal for large-scale industrial application^24–26^. Considering the above-mentioned advantages,The pre-synthesized magnetic nanoparticle (Fe_3_O_4_) displayed a good size distribution with an average size of 5–10 nm, as shown in Fig. 3A. The covalent binding method for Ch-MNPs coated with Tm-β-Glu-Tt-ChBD appeared in spherical shapes with approximately 30–60 nm in size, as presented in Fig. 3B. In comparative studies and based on the presented TEM images, the equivalent particle sizes were found to range from 22.5 to 100 nm^15,27^, with a reduction of ~40 nm in size for future applications. In Fig. 3C, FT-IR spectrum of Tm-β-Glu-Tt-ChBD-Ch-MNPs showed prominent peaks at 1062.5 and 627.16 cm^−1^ which was characteristic for stretching vibration of the C-O-C and Fe-O bond from Ch-MNPs. Moreover, the peaks observed at 1526.71 and 1382.69 cm^−1^ were corresponding to N-H deformation and C-O vibration from chitin. The characteristic peak at 1689.46 cm^−1^ revealed the presence of an amide-I region of Tm-β-Glu which confirmed the successful immobilization of Tm-β-Glu onto Ch-MNPs. The broadband at around 3320 cm^−1^ after immobilization of Tm-β-Glu-Tt-ChBD could be due to association intermolecular bonds from N-H stretching vibration with O-H stretching vibration in the β-Glu molecules^28^, which confirmed successful immobilization of Tm-β-Glu-Tt-ChBD on Ch-MNPs.

Also, through the analysis of user access strategies in the correlation between Fe_3_O_4_ MNPs synthesis with Tm-β-Glu-Tt-ChBD and specificity binding through target molecules using AutoDock software to design and correlation analysis. Consequently, MNPs can be smoothly and efficiently functionalized with thiolated molecules and carboxylic groups, which in turn, were conjugated with amino groups of such proteins, as presented in Fig. 3D. Accordingly, the high surface-to-volume ratio provided by the MNPs favored both the high binding capacity and the high catalytic specificity of the Tm-β-Glu-Tt-ChBD as well^18^.

### Optimum pH and temperature conditions on the activity of free and immobilized Tm-β-Glu-Tt-ChBD

**Figure 4:**
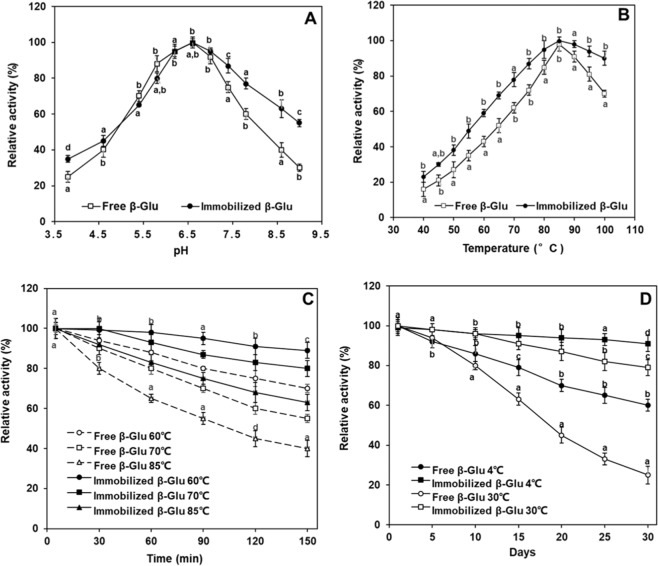
(**A**) Effect of pH on free and immobilized β-Glu, (**B**) Effect of temperature on free and immobilized β-Glu, (**C**) Thermal stability of free β-Glu and immobilized β-Glu at 60, 70, and 85 °C, respectively, (**D**) storage stability of free β-Glu and immobilized β-Glu at 4 and 30 °C, respectively. The error bars represent standard deviations from three biological replicates. Enzyme activity was measured at 90 °C, using pNPG as a substrate. The values were displayed as mean ± SD, n = 3.

The optimum pH was determined via incubating both the free and immobilized β-Glu at different pH values, as displayed in Fig. 4A. The free and immobilized enzyme exhibited maximum activity at pH 6.5. No significant change in the optimum pH was observed after immobilization. A slight shift in the optimum pH value occurred may be due to the stronger interactions between the enzyme and the carrier matrix, such as hydrogen bonding, electrostatic interactions. It is noticeable that the pH of immobilized β-Glu was significantly 1.8-fold higher than those of free β-Glu. At pH 9, the immobilized β-Glu retained 59% of relative activity, while free β-Glu was only 32%. No significant differences were observed from both types of enzymes (free and immobilized β-Glu) at pH 5. This corresponds to previously published results for Tm-β-Glu-Tt-ChBD immobilized to a variety of supports chitin beads^7,13^. Increased pH stability for immobilized-enzyme systems could be related to diffusion constraints or secondary interactions between the enzyme molecules and supports^29^. Compared to other hyperthermophilic organisms, β-Glu from *Thermatoga maritima* exhibit unique characteristics in that they can be plated with 100% efficiency at temperatures of 75–80 °C and can form colonies in 2 days on defined minimal media^7^.

The optimum temperature of the free and immobilized β-Glu was determined in the range from 40 to 100 °C, as shown in Fig. 4B. It revealed that the optimum temperature for both free and immobilized enzyme was 85 °C. The relative enzymatic activity of immobilized β-Glu was 1.5, 1.4, and 1.1-fold higher than that of free β-Glu at 60, 70, and 85 °C, respectively. Furthermore, when the temperature increased to 100 °C, the covalently Tm-β-Glu-Tt-ChBD-Ch-MNPs displayed relatively a 2.3-fold activity of 91% while the free counterpart lost about 38% of its activity. Moreover, Fig. 4B shows that, at high temperature (100 °C), the relative activity of free β-Glu was (70%) significantly lower than the immobilized β-Glu (90%) at the same conditions. Afterward, the free and immobilized enzymes were incubated in the absence of substrate at three different temperatures (60, 70 and 85 °C). The heat inactivation curves of the free and immobilized β-Glu were illustrated in Fig. 4C. At 60 °C, the activity of the immobilized and free β-Glu retained their activities about 89 and 70%, respectively after 150 min for the same incubation period. At 70 °C, the activities of the immobilized and free enzymes were retained at levels of 81 and 54%, respectively. However, the activity of Tm-β-Glu-Tt-ChBD-Ch-MNPs was higher than the free enzyme. Meanwhile, there was more decline in the catalytic properties of the free enzyme after 150 min of incubation (at 85 °C) had lost almost 59.64% of its activity. This enhancement of immobilized β-Glu enzyme in thermal stability could be due to the following reasons: (1) magnetic affinity provide suitable protection to retard the heat transfer of the Ch-MNPs structure at high temperatures, (2) the rigidity of the active conformation was improved by the cross-linkage between Tt-ChBD and Ch-MNPs.

### Storage Stability of free and immobilized Tm-β-Glu-Tt-ChBD

As presented in Fig. 4D, the storage temperature has a significant influence on the catalytic activity of the native β-Glu compared to the free β-Glu when stored at 4 °C, which retained even after 30 days (data unshown), around 61% of its original activity. Meanwhile, the similar protein retained only about 24% of its initial catalytic ability when stored at 30 °C for comparable period of time. Also, the products following the immobilization method illustrated a significant storage stability results, which was higher compared with the native β-Glu, regardless of the storage temperature. It should be also noted that the storage temperature does influence the catalytic ability of the products following immobilization. The systems stored at 4 °C, retained nearly all their original activity 91%, following the analyzed phase, while the immobilized β-Glu showed a decline in activity by approximately 12% when stored at 30 °C. Gupta *et al*. (2014) reported the activity of β-Glu immobilized on sodium alginate beads at levels of 62% and 17% after storage at 4 °C for 5 and 25 days, respectively^30^. Meanwhile, Agrawal *et al*. (2016) found that β-glucosidase immobilized on silicon oxide nanoparticles preserved 10% of its initial activity after 9 days when stored at 25 °C^31^. The results obtained in this research work revealed that the immobilization of Tm-β-Glu-Tt-ChBD on Ch-MNPs significantly support its catalytic activity compared with the free Tm-β-Glu and previously published results. This fact could be clarified owing to the protection of the three-dimensional structure of the biocatalyst, which indicates the preservation of the active centers intact, as a result of which high catalytic activity of the immobilized Tm-β-Glu-Tt-ChBD is observed^11^.

### Kinetic parameters

**Figure 5:**
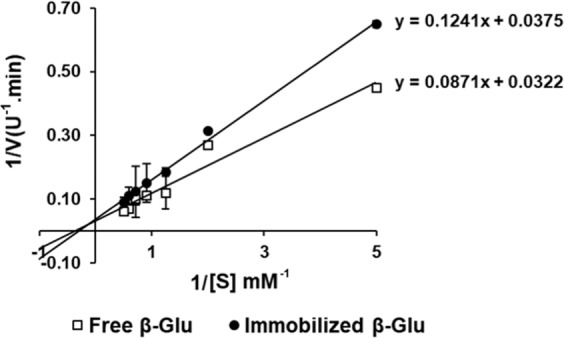
Lineweaver-Burk plot analysis of the enzymatic kinetics of free and immobilized β-Glu. The error bars represent standard deviations from three biological replicates. The different letters indicate a significant difference (p < 0.05). The values were presented as mean ± SD, n = 3.

Upon immobilization, changes in kinetic parameters (K_m_ and V_max_) were also essential to judge the success of the immobilization process. Free and immobilized Tm-β-Glu Lineweaver-Burk plots were demonstrated in Fig. 5. The constant kinetic K_m_ values for pNPG of free and immobilized Tm-β-Glu were 2.70 and 3.31 mM, respectively. However, the V_max_ value of the free Tm-β-Glu was 31.06 U mg^−1^ protein, whereas the V_max_ value of the immobilized enzyme was 26.67 U mg^−1^ protein, which can be noticed in Table 2. Çelik *et al*. found that the K_m_ and V_max_ values of the free enzyme was 2.04 mM and 5.12 U mg^−1^; but the K_m_ value increased to 5.55 mM, and the V_max_ value increased to 7.14 U mg^−1^ after the immobilization of β-Glu on MNPs while utilizing pNPG as a synthetic substrate^32^.

**Table 2:** Kinetic parameters of free β-Glu and Tm-β-Glu-Tt-ChBD-Ch-MNPs.

Enzyme	K_cat_ (s^−1^)	K_m_(mM)	V_max_ (U. min)	K_cat_/K_m_(M^−1^·s^−1^)
Free β-Glu	18.94^a^	2.70 ± 0.01^b^	31.06 ± 0.05^b^	7.01^a^
Tm-β-Glu-Tt-ChBD-Ch-MNPs	25.89^b^	3.31 ± 0.03^a^	26.67 ± 0.08^a, b^	7.82^b^

^*^The kinetic parameters were determined at pH and temperature optimal for both three different methods at pNPG concentrations from 0.2 to 2.0 mM. The values were shown as mean ± SD, n = 3.

Covalent immobilization of the enzyme on the matrix initiated a structural adjustment and reduced the affinity of the enzyme-substrate complex. Commonly, the immobilization process will improve the impediment of the enzyme towards its substrate and hence slightly increase the K_m_ value^33^ owing to the limited access to the active site of the enzyme in the cell, which happened in the immobilization procedure^34^.

### Production of GOS from free and immobilized Tm-β-Glu-Tt-ChBD

**Figure 6:**
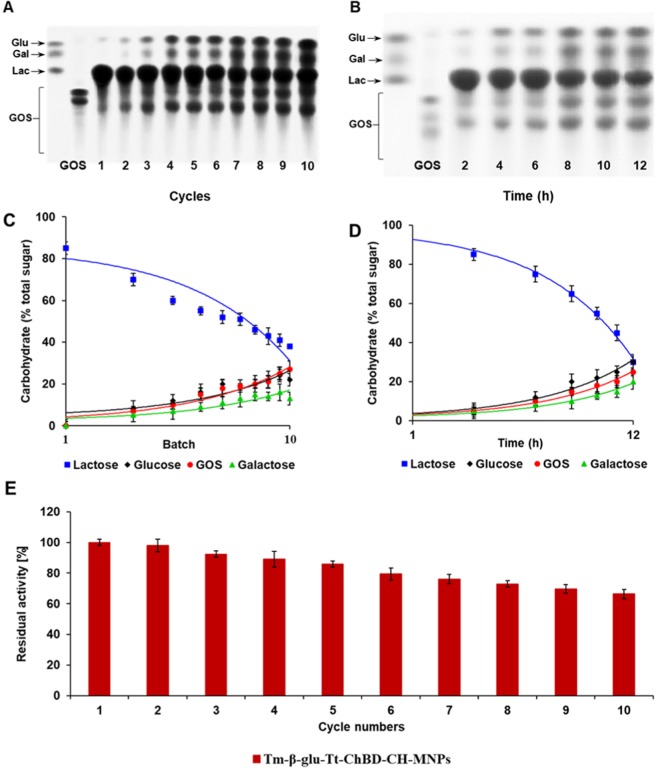
(**A**) TLC analysis of batches every 2 h of lactose conversion by the immobilized β-Glu, (**B**) TLC analysis of batch every 2 hours at different times of lactose conversion by free β-Glu, (**C**) Carbohydrate composition during lactose transformation by immobilized β-Glu determined by phenol-sulphuric acid method, (**D**) Carbohydrate composition during lactose transformation by free β-Glu determined by phenol-sulphuric acid method, (**E**) Residual immobilized β-Glu activity every 2 h. Different letters indicate the significant difference (p < 0.05), and the values were revealed as mean ± SD, n = 3. Standards used: glucose (Glc); galactose (Gal); lactose (Lac); purified GOS.

A spectrum of different GOS was produced during ten successive conversion batches of lactose at constant conditions; temperature 65 °C, pH 6.5, and 200 g L^−1^ lactose concentration^2^. The obtained GOS from each batch was analyzed by thin-layer chromatography (TLC), as shown in Fig. 6A. It was observed that the GOS formation rate increased with the decreasing in initial batches. This behavior could be due to the slow reaction between the immobilized β-Glu enzyme and lactose^35^. Conversely, the reaction was faster with the free β-Glu, and therefore, specific productivity was higher after 12 h, as illustrated in Fig. 6B. In contrast, nanoparticles immobilized β-Glu showed the weakest signal for residual lactose after the 6^th^ cycle, which reflects the higher k_cat_, K_m_ value of this variant compared with free β-Glu, as expected from the steady-state kinetics (Table 2). The highest GOS production was 19.43% when the lactose conversion reached almost 50% after five batches of reaction as displayed in Fig. 6 C, when the temperature reached 65 °C the catalytic activity of the free biocatalyst significantly decreased, suggesting that the free enzyme was unstable in these conditions owing to the denaturation of the peptide structure^36^. In the 10^th^ batch, a maximum GOS yield of 31.23% with 38.13% lactose conversion was obtained successfully, which was higher than the corresponding value that obtained only 25% of GOS produced via the native enzyme after 12 h, as shown in Fig. 6D. However, the immobilized β-Glu still retained 66% of the initial activity at the end of the operation, Fig. 6E, so there was still more room for extra batches until conquering the biocatalyst replacement point. The difference of GOS production by sequential batch with immobilized β-Glu will increase with the number of batches considered in the biocatalyst life cycle, almost 16 productive batches can be performed ultimately. The relative increase in enzymatic activity after ten batches could be explained by the mass non-loss of the enzymatic derivative during the magnetization process. These results clearly demonstrate that the magnet technology has high stability and reusability without a significant decrease in enzymatic efficiency. In addition, the technology system eliminates the need for a step to separate the enzyme from the reaction mixture.

## Materials and Methods

### Materials

Lactose, chitin, chitosan, sodium alginate, and p-nitrophenyl glycoside (pNPG) substrates were purchased from Sigma Chemical (St. Louis, MO, USA). High molecular weight chitosan (HMW-chitosan) and low molecular weight chitin (LMW-chitosan) were provided by ARDC (Agro-Industries Development Corporation, Nanjing, China). Colloidal chitin was prepared from powered chitin by the methods described by Jeuniaux^13^. Qiagen plasmid kit and Qiagen MinElute, gel extraction kit, were obtained from Gene Company (Qiagen, USA). *E. coli* JM109 (DE3) (Promega) was used for the gene expression, via the T7 RNA polymerase expression system with pET-28a plasmids (Novagen, USA)^7^. Ultra-pure water (Direct-Pure Water System, RephiLe Bioscience, Ltd., Beijing, China) with an 18.2 MΩ·cm resistivity was used through all experiments. All chemicals and reagents were used as received without further purification.

### Recombinant Tm-β-Glu and Tm-β-Glu-ChBD production and its purification

The cloning and expression of the *T. maritima* β-Glu gene (Genbank entry X74163) have been reported previously^7,11^. The genes encoding Tm-β-Glu-Tt-ChBD were obtained from pET-28a-23aa-BglA by polymerase chain reaction using PCR with the primers Tm-β-Glu-Tt-ChBD_fwd (5′- gcatccaggcacataccagcctggtgggttgggaaccgccgaacgtgccggcactgtggcagctgcagTAAGCTTGAAGGCCGCTTC-3′) and Tm-β-Glu-Tt-ChBD_rev (5′atttgtaggtgataccgttgtaggtcaccagatcaccgattttgtagtaggtgttgcttgcccattcGTCTTCCAGACCGT -3′), respectively. The expression of the recombinant β-Glu in *E.coli* JM109 (DE3), cell harvest, and purification of protein were performed as described for the Tm-β-Glu-Tt-ChBD and Tm-β-Glu^7^. SDS-PAGE analysis was used to detect the molecular mass, purity, and heat treatment of Tm-β-Glu^37^. The molecular protein weight markers 100 kDa (PageRuler Prestained Protein Ladder, Thermo scientific, 26617) were used in this work.

### Synthesis of magnetic nanoparticles (MNPs)

Two milliliters of Na_2_SO_3_ (1 M) were mixed with 3 mL of FeCl_3_ solution (2 M), then 10 mL of double-distilled water (dd-H_2_O) was added in 500 mL flask. After 1 min, 80 mL of NH_3_(aq) solution (0.85 M) was added, and the mixture was stirred magnetically for 30 min under a nitrogen atmosphere flow. The precipitate was separated by magnetic and rinsed with enough distilled water and ammonia. Lastly, the Fe_3_O_4_ product was magnetically separated and washed thrice with dd-H_2_O and ethanol to remove non-magnetic particles. The Fe_3_O_4_ MNPs were obtained by a drying oven at 60 °C for 12 h.

### Immobilization on different functionalized magnetic nanoparticles

#### Preparation of chitin-MNPs by covalent binding

Chitin solution (Ch) was prepared by stirring the mixture of 250 mg of chitin with 12.5 mL of (0.1 M) phosphoric acid at 4 °C for 24 h. Afterward, the Ch beads were washed repeatedly with dd-H_2_O and centrifuged at 10,000 rpm for 10 min and decantation to remove the unsuspended particles. After that, the pH was adjusted to 7 by using (6 M, NaOH), and the solution was re-centrifuged, then the supernatant was collected. Before usage, the Ch beads were washed three times with sodium phosphate buffer (50 mM, pH6.5) and re-suspended in the same buffer. MNPs were prepared by mixing an aqueous solution of Ch (0.2 mg mL^−1^) and the previously prepared colloidal suspension of MNPs in the ratio of 2:1(w/w). The reaction mixture was sonicated in an ultrasonic bath for 8 h at 40 °C. Then Ch coated MNPs (Ch-MNPs) were washed with dd-H_2_O by magnetic separation to remove the free Ch. The other two materials, chitosan (Cs) and sodium alginate (SA) solutions, were performed and achieved Cs-MNPs and SA-MNPs, respectively according to previous studies^38,39^.

#### Immobilization of Tm-β-Glu-Tt-ChBD on Ch-MNPs, Cs-MNPs, and SA-MNPs

The Ch-MNPs, Cs-MNPs, and SA-MNPs (10 mg mL^−1^) were immersed in Tm-β-Glu-Tt-ChBD (1 mg mL^−1^) with sodium acetate buffer (50 mM, pH 5.0). All mixtures were incubated at 4 °C under mild shaking (100 rpm) for 12 h. Later, the non-covalently adsorbed protein was removed after that by a thorough washing of the coated MNPs with dd-H_2_O and sodium acetate buffer solution via a magnetic response Fig. 2C. The immobilized and non-immobilized nanocomplex was assessed by SDS-PAGE, activity assay, and protein concentration measurements. The immobilization efficiency and immobilization yield were also calculated based on the earlier optimized method^40^.

### Protein determination and enzyme assay

Total protein concentration was determined by the Bradford method using bovine serum albumin (BSA) as standard^41^. The activity of free Tm-β-Glu, Tm-β-Glu-Tt-ChBD-Ch-MNPs, Tm-Glu-Cs-MNPs, and Tm-β-Glu-SA-MNPs was determined using the chromogenic substrate 4-Nitrophenyl-β-D-glucopyranoside (pNPG). Similarly, the enzyme activity assay was carried out in 10 μL (23.17 mg mL^−1^) of the suitably diluted enzyme in 180 μL of 50 mM potassium phthalate buffer (PPB, pH 6.2) and 10 μL of substrate solution (40 mM pNPG)^7^. After incubation at 90 °C for 5 min, the reaction was stopped by directly adding 600 μL of sodium carbonate (1 M) to the reaction mixture. A standard curve was prepared using p-nitrophenol (pNP). The amount of pNP generated during the reaction was detected using a UV–vis spectrophotometer (AT-UV-2900) at λmax 405nm^8^. The enzyme activity was then calculated by using the following equation [enzyme activity (EA) = (Units/ml)]. One unit of pNPG activity was defined as the amount of enzyme releasing 1 µmol of pNP per minute.

### Characterization of free and immobilized Tm-β-Glu-Tt-ChBD enzymes

Fourier transform infrared spectroscopy (FT-IR) was performed to investigate the shifting in the absorbance owing to the chemical binding as immobilization Tm-β-Glu-Tt-ChBD on Ch-MNPs. Among that, the sample was recorded employing a Tensor 27 infrared spectrometer (Bruker, Germany) from KBr disc in the range of 4000–400 cm^−1^. The morphology of the immobilized Tm-β-Glu-Tt-ChBD particles was investigated using transmission electron microscopy (TEM) (Hitachi H-7650, Japan). The sample was firstly re-dispersed in ethanol via ultrasonic, and then 10 μL was carefully dropped over the copper grid and kept for 30 min to dry totally.

### Optimum pH and temperature of the free and immobilized Tm-β-Glu-Tt-ChBD enzymes

The optimum pH and temperature of Tm-β-Glu-Tt-ChBD were checked in range (4.0–9.0) and (35–100 °C), respectively, for both enzymes (free and immobilized). The optimum pH for immobilized and free β-Glu activity was determined using standard assay conditions in sodium acetate buffer at varying pH (pH 4.0–9.0, 20 mM). The optimal temperatures for immobilized and free Tm-β-Glu activities were determined under optimal pH and incubated at various temperatures (35–100 °C). Meanwhile, the thermal stability study of both the Tm-β-Glu-Tt-ChBD immobilized, and free enzyme were performed by pre-incubating the enzyme at different temperatures ranging from 60 to 85 for 150 min at the obtained optimum pH. The activity of the free and immobilized β-Glu was determined by pNPG as a substrate.

### Determination of the kinetic constants

The kinetic constants, Michaelis constant (K_m_) and maximum velocity (V_max_) values of the free and the immobilized Tm-β-Glu-Tt-ChBD were determined by measuring the initial rates of the reaction with pNPG from 0.2 to 2.0 mM in sodium acetate buffer (50 mM, pH 6.5) at 65 °C. Then, the K_m_ and V_max_ values were calculated from the Lineweaver-Burk plot.

### Effect of storage on Tm-β-Glu-Tt-ChBD activity of immobilized

The effect of storage stability of both the immobilized and free enzyme was tested when stored at 4 °C and 30 °C in PBS (pH 6.5) for 30 days. The activity of each enzyme was measured at intervals of 1, 5, 10, 15, 20, 25, and 30 days. The activity of the free and immobilized β-Glu was determined by pNPG as a substrate.

### Screening of synthesis GOS TLC and phenol-sulphuric acid method

Synthesized GOS products were screened by silica gel thin-layer chromatography (TLC) to investigate the activity of the free and immobilized Tm-β-Glu. A volume of 5 mg mL^−1^ immobilized Tm-β-Glu was mixed with 1 mL lactose (200 g L^−1^) in sodium phosphate buffer (50 mM, pH 6.6) containing (1 mM) MgCl_2_ and incubated with shaking at 150 rpm and 65 °C. The synthesis was carried out for ten consecutive batches every 2 h. The transglycosylation reaction of lactose was emptied after each batch, and the biocatalyst was recovered by magnetic separation techniques. The obtained synthesis GOS was determined by TLC and phenol-sulphuric acid method. Similarly, lactose was hydrolyzed in a batch every 2 h using free Tm-β-Glu at different times. The samples were placed on cold water at 4 °C for 5 min and diluted 1:10, followed by loading of 1 μL sample on a TLC plate. Later, samples were applied on the plates and placed in the eluent n-butanol/2-Propanol/water (1:4:1). Visualization of the separated carbohydrates after drying was carried out by using a blow dryer and was visualized by spraying a 20% sulfuric acid-methanol solution, followed by incubation at 95 °C for approximately 1 min.

Regarding the estimation of GOS by the phenol-sulphuric acid method, 2.0 mL from each batch were chromatographic on a silica gel plate (Dieselgel 60: Merck Co., Berlin, Germany). Accordingly, samples were separated from the supernatant by filtration and rinsed with dd-H_2_O for three times. The products of the enzymatic reaction, including glucose, galactose, lactose, and GOS products were measured by the phenol-sulphuric acid method using glucose as standard^35^. Sample solution (0.5 mL, 100 g mL^−1^) was mixed with 0.5 mL phenol solution (6%) and 2.5 mL sulfuric acid. After 20 min, the absorption at 490 nm was measured via a spectrophotometer^42^. Glucose in range (1 mg mL^−1^) was used as a standard, the measurements were repeated in triplicate.

### Statistical analysis

All measurements were done in triplicate, and data were reported as mean ± standard deviation (SD). Analysis of variance (ANOVA) accompanied by Duncan test using SPSS software (version 16.0 for Windows, SPSS lns., Chicago) was conducted to identify the significant differences between samples (p < 0.05).

## Conclusion

In this study, β-glucosidase from *Thermotoga maritima* was immobilized on three different biopolymers, for instance, chitin, chitosan, and sodium alginate via Fe_3_O_4_ magnetic nanoparticles. Chitin exemplified the most strong binding affinity by fusing target proteins with a novel thermostable chitin-binding domain. Among the SDS-PAGE and enzyme kinetics, chitin was also the highest enzyme binding through the chitin-restricting space. Also, the results showed a substantial increase in GOS production after the second cycle of passing with a maximum GOS yield of 31.23%. The immobilized β-glucosidase on chitin nanoparticles keet 79% activity and produce 31.23% GOS after ten repeated cycles when compared with that of the free enzyme, demonstrating high efficiency and reusability. Remarkably, the magnetic separation technique was successfully employed for the reuse of the immobilized β-glucosidase for repetitive batch-wise GOS without significant loss of activity. After optimizing the recycling ability of the enzyme, the magnetic immobilized enzymes bioconjugates can be potentially employed in the manufacturing of a variety of commercially useful pharmaceutical metabolites. Based on the literature survey and several successful examples, it can be inferred that the use of MNPs provides a critical approach for enzyme immobilization with a definite broad perspective in enzyme catalysis and other fields.

## Supplementary information

Supplementary information.

Supplementary  video.
