# Supplementary material for: Immobilization of β-Glucosidase from Thermatoga maritima on Chitin-functionalized Magnetic Nanoparticle via a Novel Thermostable Chitin-binding Domain
Source: Sci Rep. 2020 Feb 3;10:1663. doi: 10.1038/s41598-019-57165-5 (PMC6997207; doi:10.1038/s41598-019-57165-5)
Supplement: Supplementary file 1 — Supplementary information. [file 41598_2019_57165_MOESM1_ESM.docx]

**Immobilization of β-Glucosidase from *Thermatoga maritima* on Chitin-functionalized Magnetic Nanoparticle via a Novel Thermostable Chitin-binding Domain**

**Fawze Alnadari, ^1,6^**^†^ **Yemin Xue,** **^1^**^†^ **Liang Zhou, ^1^ Yahya S. Hamed,** ^7^ **[Mohamed Taha](https://www.sciencedirect.com/science/article/pii/S0016706117316919#!)**^4,5^ **and Mohamed F. Foda ^2,3,^**^4^**^*^**

^1^ Department of Food Science and Engineering, School of Food Science and Pharmaceutical Engineering, Nanjing Normal University, Nanjing 210023, Jiangsu, P.R. China.

^2^ State Key Laboratory of Agricultural Microbiology, College of Science, Huazhong Agricultural University, Wuhan 430070, P. R. China.

^3^ State Key Laboratory of Agricultural Microbiology, College of Veterinary Medicine, Huazhong Agricultural University, Wuhan 430070, P. R. China.

^4^ Department of Biochemistry, Faculty of Agriculture, Benha University, Moshtohor, Toukh13736, Egypt.

^5^ Centre for Environmental Sustainability and Remediation, RMIT University, Bundoora, Melbourne, VIC 3083, Australia

^6^ Department of Food Science and Technology, Faculty of Agriculture, Sana'a University, Sana'a, Yemen.

^7^ Food Technology Department, Faculty of Agriculture, Suez Canal University, Ismailia, 41522, Egypt.

† **Contributed equally to this work**

**ــــــــــــــــــــــــــــــــــــــــــــــــــــــــــــــــــ**

**^*^Correspondence:** Dr. Mohamed Frahat Foda, **PI**, State Key Laboratory of Agricultural Microbiology, College of Science and College of Veterinary Medicine. Address: Huazhong Agricultural University, Wuhan 430070, P.R. China; **Tel:** +86-13720279115. **E-mail:** [m.frahat@fagr.bu.edu.eg](mailto:m.frahat@fagr.bu.edu.eg)


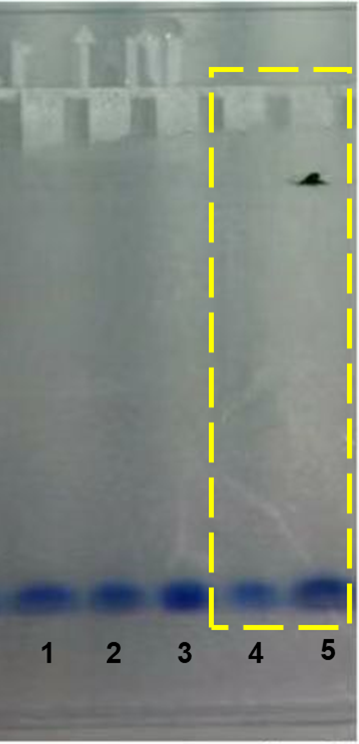


**Supplementary Figure S1.** **Uncropped gel image of all gel in Fig. 2A:** Tm-β-Glu-Tt-ChBD-Chitin-MNPs

**
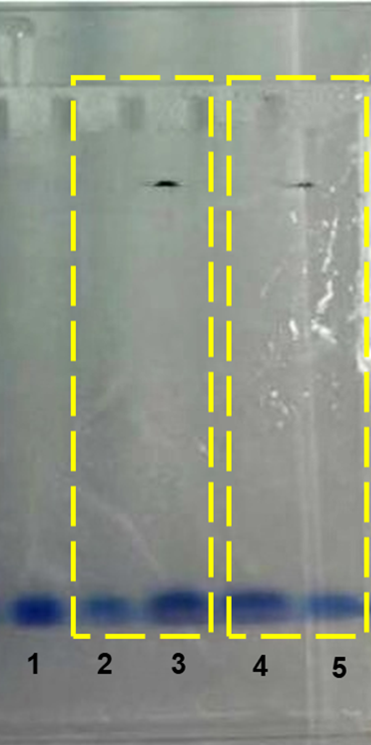
**

**Supplementary Figure S2. Uncropped gel image of all gel in Fig. 2B:** Tm-β-Glu-Sodium alginate-MNPs and Tm-β-Glu-Chitosan-MNPs
